# A Challenging Case of Viral Pneumonia in the COVID-19 Pandemic Era

**DOI:** 10.7759/cureus.59360

**Published:** 2024-04-30

**Authors:** Yusuf Ziya Şener, Ahmet Emre Gultekin, Akif Can Guler, Ugur Canpolat, Sehnaz Alp

**Affiliations:** 1 Cardiology Department, Hacettepe University Faculty of Medicine, Ankara, TUR; 2 Internal Medicine Department, Hacettepe University Faculty of Medicine, Ankara, TUR; 3 Infectious Diseases Department, Hacettepe University Faculty of Medicine, Ankara, TUR

**Keywords:** valganciclovir, pneumonia, catheter ablation, cytomegalovirus-cmv, covid 19

## Abstract

Cytomegalovirus (CMV) is a DNA virus that can cause widespread, severe infection in immunocompromised patients. While CMV usually leads to a subclinical infection in immunocompetent individuals, it can rarely cause severe disease in this population. The SARS-CoV-2 virus is an RNA virus and part of the *Coronaviridae* family. SARS-CoV-2 led to the COVID-19 (coronavirus disease 2019) pandemic. Even though COVID-19 usually presents with signs and symptoms of upper respiratory tract infection in younger adults, viral pneumonia, cytopenia, and neurological symptoms become more apparent with increasing age. Herein, we describe an immunocompetent 73-year-old female patient in whom oxygen demand and pancytopenia developed during hospitalization for post-ablation inguinal access site infection. The thorax CT revealed viral pneumonia, but two subsequent SARS-CoV-2 polymerase chain reaction (PCR) tests and a viral respiratory multiplex PCR panel were negative. The CMV viral load was high in the blood sample, and the patient responded to valganciclovir treatment. Although SARS-CoV-2 should be evaluated in patients with viral pneumonia and cytopenia, other viral etiologies mimicking SARS-CoV-2 infection, such as CMV, should not be overlooked in the era of the COVID-19 pandemic.

## Introduction

SARS-CoV-2 is an RNA virus belonging to the *Coronaviridae* family. It first appeared in China, causing the disease later named COVID-19 (coronavirus disease 2019), which had become a pandemic in a few months worldwide. Fever, cough, shortness of breath, diarrhea, myalgia, and fatigue are the main symptoms of the disease. The most frightening manifestations of the illness are viral pneumonia and acute respiratory distress syndrome, which can result in respiratory failure and death [1].

Cytomegalovirus (CMV) is a DNA virus that typically infects the majority of individuals during their fourth decade. Immunocompetent individuals who are infected with CMV usually experience either no symptoms or a mild disease. However, in immunocompromised patients, CMV can cause severe life-threatening infections. CMV pneumonia is a rare disease in immunocompetent individuals and is reported in only a few cases in the literature [2]. The rate of CMV infection in immunocompetent patients admitted to the ICU was reported at 27%, and CMV infection was established to be related to increased mortality rates [3].

Due to the COVID-19 pandemic, viral pneumonia due to other etiologies can easily be overlooked because of the rising awareness of the SARS-CoV-2 infection. Thus, we aimed to present a case report of a 73-year-old, apparently immunocompetent female who was diagnosed with CMV pneumonia and for whom COVID-19 was excluded, although she presented with typical signs and symptoms of SARS-CoV-2 infection.

## Case presentation

**Figure 1:**
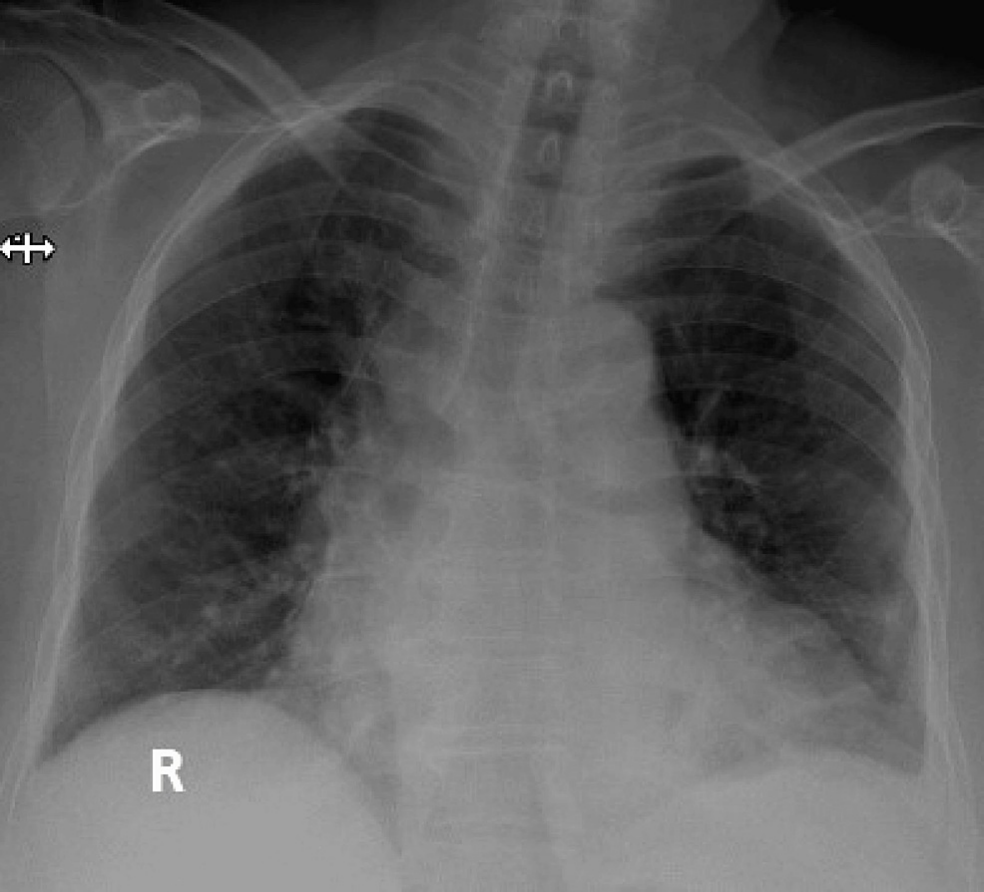
The chest X-ray shows patchy bilateral infiltrations located mainly in the lower and lateral zones.

A 73-year-old woman with a known case of hypertension and diabetes mellitus was admitted to the emergency room with a fever and purulent discharge from her right inguinal wound after catheter ablation in March 2020. Past medical history revealed a surgical pericardial window for recurrent pericardial effusion three months ago, and thereafter, radiofrequency catheter ablation for atrial tachycardia refractory to antiarrhythmic drug treatment was performed one month before her admission. She had been diagnosed with diabetes and hypertension 10 years ago, and she did not have any target organ damage. She was a non-smoker, and she claimed not to use any substances. Her medications on admission were colchicine (0.5 mg, BID, for pericardial effusion), metformin (1000 mg, BID, for diabetes), sotalol (40 mg, BID, for atrial fibrillation), and ramipril hydrochlorothiazide (10/12.5 mg, QD, for hypertension). Her physical examination was normal (body temperature: 38 °C, blood pressure: 120/75 mmHg, respiratory rate: 16/min, SaO_2_: 91%), except for fever with warming and redness at the inguinal catheter access site. A complete blood count yielded neutrophil-dominated leukocytosis (11.4 × 10^9^/L) with mild thrombocytopenia (105 × 10^9^/L) and anemia (10.2 g/dL). Kidney and liver function tests were within normal limits. The COVID-19 outbreak had just reached our country by the time she was admitted to our hospital, and she did not have any symptoms compatible with the SARS-CoV-2 infection or any contact history with a potential case of COVID-19. The ampicillin-sulbactam (IV: 1000 mg/500 mg, QID) was administered, and the patient was transferred to an inpatient ward. The culture of the discharge from the inguinal wound revealed no bacterial growth. During follow-up, pancytopenia (hemoglobin: 9.5 g/dL, leukocyte count: 3.8 × 10^9^/L, and platelet count: 74 × 10^9^/L) developed, and the patient complained of dyspnea on the fifth day of ampicillin-sulbactam treatment. Her blood gas analysis (pH: 7.35, sO_2_: 87%, pO_2_: 50 mmHg, pCO_2_: 42 mmHg) showed that she had hypoxemic respiratory failure. Her chest X-ray demonstrated patchy bilateral infiltrations located in the lateral zones (Figure 1).

**Figure 2:**
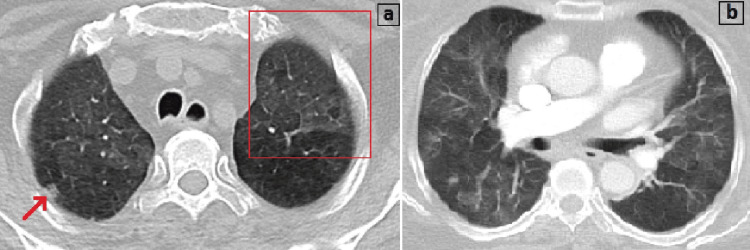
(a) The CT image shows a small nodule (red arrow) and a crazy-paving sign (red square), and (b) bilateral ground-glass opacities consistent with viral pneumonia.

The thorax CT was consistent with viral pneumonia due to the presence of bilateral ground-glass opacities and a crazy-paving sign (Figure 2).

The CT excluded pulmonary embolism. Ampicillin-sulbactam was switched to piperacillin-tazobactam (IV: 4 g/500 mg, QID) due to increased oxygen demand and extended hospital stay. The patient was isolated because of the suspicion of SARS-CoV-2 infection. Two subsequent SARS-CoV-2 polymerase chain reaction (PCR) tests at least 24 hours apart, along with a multiplex PCR assay to detect other respiratory viruses (influenza A and B, adenovirus, enterovirus, parainfluenza viruses, respiratory syncytial virus), were studied from the nasopharyngeal swab, but the results were negative. Even though she did not have any known particular predisposing conditions leading to immunocompromisation except being elderly, bronchoalveolar lavage was offered for diagnoses of potential CMV pneumonia. However, the patient refused the procedure, and plasma CMV viral load was assessed.

Plasma CMV viral load was detected as 6517 copies/mL, and CMV serology was compatible with previous exposure to CMV (CMV IgM: 0.34 TU/mL (negative), CMV IgG: 206 AU/mL (positive)). There were no other signs of CMV infection, including jaundice, hepatomegaly, and retinitis. Valganciclovir 900 mg BID PO was administered with the diagnosis of CMV pneumonia. The patient's oxygen requirement reduced gradually and completely resolved on the fourth day of her follow-up. Pancytopenia improved, and plasma CMV viral load decreased to 103 copies/mL on the fifth day of the valganciclovir treatment. The duration of piperacillin-tazobactam treatment was completed to 10 days. Her inguinal wound was also healed, and she was discharged uneventfully.

## Discussion

CMV infections are usually seen in immunocompromised patients, but although it is a rare condition, CMV may also cause severe infections in immunocompetent individuals [3]. CMV pneumonia is one of the most severe forms of CMV infection, and it is diagnosed by serology, culture, histology, or nucleic acid amplification tests such as PCR [2]. A serological diagnosis is established by demonstrating the presence of IgM antibodies or increasing titers of IgG antibodies in consecutive tests. CMV PCR is an essential diagnostic tool, and it is more valuable to detect it in bronchoalveolar lavage specimens. Blood CMV PCR is usually negative in immunocompetent patients with CMV pneumonia but may have diagnostic value if positive [4]. Due to being a rare condition in immunocompetent patients, a definite CMV pneumonia diagnosis requires histological evaluation. The CMV virus may also be detected in lung tissue as a bystander without causing the infection, but recognizing signs such as intranuclear inclusion bodies, which are specific for CMV infection, are valuable findings for diagnosis [5].

Imaging is helpful for the diagnosis of CMV pneumonia. Thorax CT may reveal several suggestive findings. Despite the frequency and severity of findings that differ between immunocompetent and immunocompromised patients, common results are mixed alveolar-interstitial infiltrates such as ground-glass opacity, consolidation, bronchiectasis, and interstitial reticulation. It is also reported that there may be small nodules in patients with CMV pneumonia, which correspond histologically to areas of hemorrhagic nodules or organizing pneumonia [6]. Main CT findings in CMV pneumonia include lobar consolidation, bilateral ground-glass opacities, irregular reticular thickening, and the halo sign, which is defined as the presence of miliary or small nodules with associated areas of ground-glass opacities [7]. SARS-CoV-2 pneumonia may cause several CT findings, including ground-glass opacities, reticular thickening, air bronchogram, a subpleural curvilinear line, fibrosis, and nodules. The crazy-paving sign is also known as the cobble-stone sign, and it defines the presence of reticular septal thickening with superimposition on a ground-glass opacity background. In the era of the COVID-19 pandemic, the crazy-paving sign can be interpreted as a radiological finding for SARS-CoV-2 infection. However, it should be kept in mind that it can also appear due to other infectious diseases, such as pneumocystis pneumonia and pulmonary aspergillosis [8,9].

There are some differences in the course of CMV infection between immunocompetent and immunocompromised patients. It is reported that immunocompetent patients with CMV infection are older and have more ICU admissions than immunocompromised cases. Interestingly, the incidence of negative results for blood CMV PCR and the mortality rate are higher in immunocompetent patients than in their immunocompromised counterparts. The clinical picture may also be different, and gastrointestinal involvement usually presents with bleeding in immunocompetent cases, while diarrhea and abdominal pain are the main symptoms in immunocompromised patients. However, the treatment approach is similar for both groups of patients [10].

CMV infections are treated with ganciclovir or its analog valganciclovir. Valganciclovir 900 mg BID is typically administered in 4-21 days, as stated in the literature [2]. In our patient, we completed the treatment in 21 days despite her oxygen demand disappearing on the fourth day of the treatment.

This case demonstrates the importance of considering other viral etiologies, such as CMV, as a differential diagnosis in cases of viral pneumonia, even in immunocompetent individuals. This is particularly important for those who do not improve with antimicrobial treatment and have negative SARS-CoV-2 results during the COVID-19 pandemic.

## Conclusions

To conclude, SARS-CoV-2 infection mainly affects the respiratory tract, and the most common cause of morbidity and mortality is due to pneumonia-related manifestations. CMV is an opportunistic infectious agent, but rarely, it may cause infections in immunocompetent patients. CMV infection should be considered in patients with viral pneumonia and pancytopenia in the era of the COVID-19 pandemic, especially when the SARS-CoV-2 tests are negative.

## References

[1] Zhang R, Ouyang H, Fu L (2020). CT features of SARS-CoV-2 pneumonia according to clinical presentation: a retrospective analysis of 120 consecutive patients from Wuhan city. Eur Radiol.

[2] Waqas QA, Abdullah HM, Khan UI, Oliver T (2019). Human cytomegalovirus pneumonia in an immunocompetent patient: a very uncommon but treatable condition. BMJ Case Rep.

[3] Li X, Huang Y, Xu Z, Zhang R, Liu X, Li Y, Mao P (2018). Cytomegalovirus infection and outcome in immunocompetent patients in the intensive care unit: a systematic review and meta-analysis. BMC Infect Dis.

[4] Cunha BA, Pherez F, Walls N (2009). Severe cytomegalovirus (CMV) community-acquired pneumonia (CAP) in a nonimmunocompromised host. Heart Lung.

[5] Cunha BA (2010). Cytomegalovirus pneumonia: community-acquired pneumonia in immunocompetent hosts. Infect Dis Clin North Am.

[6] Moon JH, Kim EA, Lee KS, Kim TS, Jung KJ, Song JH (2000). Cytomegalovirus pneumonia: high-resolution CT findings in ten non-AIDS immunocompromised patients. Korean J Radiol.

[7] Franquet T (2011). Imaging of pulmonary viral pneumonia. Radiology.

[8] Ye Z, Zhang Y, Wang Y, Huang Z, Song B (2020). Chest CT manifestations of new coronavirus disease 2019 (COVID-19): a pictorial review. Eur Radiol.

[9] Rai DK, Kumar P (2023). Crazy paving pattern due to invasive pulmonary aspergillosis: a rare case report with literature review. Med J Dr D Y Patil Vid Univ.

[10] Chaemsupaphan T, Limsrivilai J, Thongdee C, Sudcharoen A, Pongpaibul A, Pausawasdi N, Charatcharoenwitthaya P (2020). Patient characteristics, clinical manifestations, prognosis, and factors associated with gastrointestinal cytomegalovirus infection in immunocompetent patients. BMC Gastroenterol.

